# Spatial Analysis on the Role of Multi-Dimensional Urbanizations in Carbon Emissions: Evidence from China

**DOI:** 10.3390/ijerph19095315

**Published:** 2022-04-27

**Authors:** Mingyuan Guo, Shaoli Chen, Yu Zhang

**Affiliations:** College of Management and Economics, Tianjin University, Tianjin 300072, China; chestnut78@tju.edu.cn (S.C.); zhangyu22@tju.edu.cn (Y.Z.)

**Keywords:** carbon emissions, population urbanization, economic urbanization, consumption urbanization, living urbanization, spatial Durbin model

## Abstract

Using the panel data of 30 provinces in China from 1997 to 2015, this paper studies the impacts of urbanization on carbon emission. We use the entropy weight method to measure the weight of the indicator to evaluate four-dimensional urbanizations, including population, economic, consumption and living urbanization. In addition, we investigated the spatial correlation of carbon emissions, taking the spatial differences into consideration. The spatial Durbin model is finally selected to analyze the impacts of urbanizations on carbon emission. The conclusions are: Firstly, from the results of the panel data model, the four dimensions of urbanization all play a significant role in promoting carbon emissions in the whole regions. However, in eastern China, central China and western China, four dimensions of urbanization have different impacts on carbon emissions. Secondly, from Moran’s I of carbon emissions from 1997 to 2015 in China, we conclude that carbon emissions in China present a significant spatial aggregation. Thirdly, from the results of spatial econometrics model, population urbanization only promotes local carbon emissions. Economic urbanization and consumption urbanization promote local carbon emissions and reduce carbon emissions in its neighboring provinces. Living urbanization promotes both local carbon emissions and its neighboring provinces’ carbon emissions. This paper proposes some recommendations for the carbon emission decreasing during urbanization. First, establishment and improvement of coordination mechanisms and information sharing mechanisms across regions should also be considered. Second, control population growth reasonably and optimize population structure in order to achieve an orderly flow and rational distribution of the population. Third, the assessment mechanism of the local government should include not only economic indicators but also other indicators.

## 1. Introduction

Urbanization is the inevitable result of the development of industrialization to a certain stage. Since its reform and opening up, China’s urban construction has been increasingly improved and urban agglomerations exist in many regions of China. The area of urban built-up areas in China has gradually expanded. Infrastructure systems such as housing conditions, urban transportation, water supply, heat and power, greening, environmental sanitation, and telecommunications have been continuously improved. In recent years, China’s urbanization process has been obvious. The role of population mobility has been strengthened, but the problems associated with urbanization have become more and more prominent [1,2]. Many regions in China have an incomplete understanding of urbanization, which focus on urban construction and ignore regional coordination [3]. Some regions focus on scale expansion while neglecting effective resource protection [4]. The impact of urbanization on environmental pollution is different in countries and regions with different levels of development [5]. There is a serious “polarization” trend in China’s urbanization. The population and space of some cities have expanded rapidly. The number and proportion of large cities have been increasing, while the number and proportion of small and medium-sized cities are decreasing.

All countries are paying attention to the coordinated development of economy and ecological environment [6]. However, urban environmental pollution has become increasingly prominent [7,8]. Although the government’s investment in urban infrastructure has increased year by year [9,10], urban infrastructure cannot match resources and environment. China’s urbanization is accompanied by high carbon emissions. China is facing enormous pressure of carbon emissions reduction in the process of urbanization. In 2015, Chinese government committed that China’s carbon emissions per unit of GDP in 2030 will decline by 60% to 65% compared with those of 2005 [11]. It is very important to solve the problems of carbon emissions and urbanization in China. The Chinese government should fully understand the impact of urbanization on carbon emissions and implement the corresponding policies according to different situations.

The exploration of the effects of urbanization on carbon emissions from different perspective has appealed to scholars across the world. Many studies do research from population urbanization [12,13,14,15,16]. Some scholars studied the impact of urbanization on carbon emissions from multi-dimensional urbanization [17,18,19,20,21]. However, most scholars’ studies have not considered the spatial dependence between carbon emissions and urbanization. If spatial dependencies are ignored, the regression results may be biased. Secondly, China has experienced a rapid urbanization process. Affected by the differences in economic and social development, each region in China is in a different stage of urbanization. It is very important to study the impact of multi-dimensional urbanization on carbon emissions considering spatial dependence between carbon emissions and urbanization. Our research aims to formulate effective low-carbon strategies in China from the perspective of urbanization. 

In order to better analyze the impact of urbanization on carbon emissions in China, this paper measures urbanization from four dimensions, including population urbanization, economic urbanization, consumption urbanization and living urbanization. In addition, this paper uses both the panel data model and the spatial econometric model to study the impacts of four-dimensional urbanizations on carbon emissions from 1997 to 2015 in China. We aim to find whether different dimensional urbanizations have different impacts on carbon emissions in China and different regions of China using the panel data model. In addition, we tend to study whether carbon emissions in China present a significant spatial aggregation. By using spatial econometrics model, we aim to find the direct, indirect, and total effects of four-dimensional urbanizations on local carbon emissions and its neighboring provinces’ carbon emissions. Finally, we provide effective policy suggestions for reducing carbon emissions in China.

Our contributions in this paper are as follows. Firstly, previous studies are mostly based on one dimension of urbanization, without taking multi-dimensional urbanization into account. To study the influence of urbanization on carbon emissions comprehensively, this paper measures urbanization from four dimensions. Secondly, we utilize entropy weight method to measure the weight of indicators to evaluate four-dimension urbanizations. Thirdly, we take spatial dependence into consideration and adopt spatial econometric models to study the impact of multi-dimensional urbanization on carbon emissions.

The paper is designed as below. Section 2 is the literature review. Section 3 details the methodology and data source. Section 4 describes the findings of the empirical model. Section 5 gives conclusions and suggestions. 

## 2. Literature Review

### 2.1. Urbanization Indicators

#### 2.1.1. One-Dimensional Urbanization Indicator

Urbanization contains rich connotations. Population urbanization represents the process of populations in rural areas moving to urban areas. From the perspective of economics, economic urbanization represents a change in economic structure. Living urbanization represents the improvement of urban infrastructure. 

At present, there are many studies on population urbanization [12,13,14,15,16], because population urbanization is easy to obtain and calculate. Some studies not only adopted the proportion of urban population as an indicator of population urbanization, but also took the structure of urbanization into account [22].

#### 2.1.2. Multi-Dimensional Urbanization Indicator

Using a single indicator to measure urbanization does not fully consider various aspects of urbanization. Therefore, some scholars used comprehensive indicators to measure urbanization, which take population, economic structure, lifestyle, and land usage into account. Urbanization was a complex system that integrated population, land, and economy [17,18]. Some scholars constructed multi-dimensional urbanization using four indicators, including Zipf’s coefficient, spatial Hirschman–Herfindahl index, urban priority index, and spatial Gini coefficient [19]. Some scholars constructed urbanization from the perspective of population urbanization, economic urbanization, consumption urbanization, and living urbanization [23]. 

### 2.2. Urbanization’s Impacts on Carbon Emissions

#### 2.2.1. Population Urbanization’s Impacts on Carbon Emissions

Some researchers conclude that population urbanization increases carbon emissions [24,25]. However, some researchers have reached the opposite consequence [26]. They conclude that in the process of urbanization, energy expenditure and carbon emissions were alleviated because of the optimized allocation of resources. The evolution of population urbanization in China reduces carbon emissions by increasing energy efficiency [27]. 

#### 2.2.2. Economic Urbanization’s Impacts on Carbon Emissions

Economic urbanization is defined as the transformation of the economic structure. Due to limited resources, the environmental problems faced by cities in the low development stage are often related to poverty, such as inadequate sanitation and a lack of water supply. Urban wealth increases with the increase of manufacturing activities, which causes water pollution, air pollution, and industrial pollution. These problems also decrease with the continuous urban development, due to the enrichment of environmental laws and regulations, technological development, and changes in economic structure. As society gradually realizes the importance of environmental sustainability, the influence of economic development on the environment is reduced by scientific innovation, urban agglomeration, and industrial restructuring [28,29,30]. China’s economic rise has attached to huge shifts in the industrial structure. Industrial structure has diverse influences on carbon emissions at diverse stages [31]. Industrial structure plays a substantial role in promoting carbon emissions [32,33].

#### 2.2.3. Consumption Urbanization’s Influence on Carbon Emissions

Many scholars also study the influence of consumption urbanization on carbon emissions. As wealth increases, there are more and more environmental issues related to consumption. The lifestyles of rich cities are more resource-intensive than those of poor cities. With the augmentation of urban wealth, residents’ requirement for urban infrastructure, transportation conditions, and resources also increase. Therefore, the environmental issues related to consumption have become more prominent. The influence of urbanization on carbon emissions is positive for all families, but it is most apparent for middle-income families [34,35,36]. Income has impacts on carbon emissions in OECD countries [37]. 

Consumer’s consumption, including private cars, housing, etc., leads to 45% to 55% of total energy consumption [38]. Some researchers analyzed the influence of changes in the resident consumer’s behavior on carbon emissions using Korean data from 1985 to 1995. They found direct residential energy consumption and the demand for high-carbon consumer goods increased greenhouse gas emissions [39]. The influence of economic development, expenditure structure, urban–rural structure, and other factors on carbon emissions of urban areas is larger than that of rural areas [40]. Engel’s coefficient, private car ownership, and the number of internet users all have impacts on carbon emissions [41].

#### 2.2.4. Living Urbanization’s Impacts on Carbon Emissions

Living urbanization includes the change in land utilization patterns and the change in urban spatial patterns. In general, the increase in urban density enables urban infrastructure to fully realize economies of scale and reduce energy consumption [42]. For example, the aggregation of public facilities increases the economies of scale of these facilities. In addition, a compact urban space also reduces residents’ travel distance and reduces energy consumption. However, excessive urban density increases urban environmental pollution. For example, excessive urban density causes traffic congestion and releases more carbon dioxide. The compact cities needed adequate infrastructure; otherwise, urban environmental problems tended to be worse [43]. 

Some researchers also analyzed the relationship between CO_2_ emissions and land utilization. The compact urban space reduced CO_2_ emissions [44]. Urban compactness was positively related with urban carbon dioxide economic efficiency [45]. Living urbanization has an apparent inhibitory influence on carbon emissions [46].

## 3. Methodology and Data Source

### 3.1. Panel Data Model

The panel model which studies the impacts of four-dimensional urbanizations on carbon emissions is defined as follows:

(1) $$\ln CE_{i,t}=c+\beta_{1}\ln PURB_{i,t}+\beta_{2}\ln EURB_{i,t}+\beta_{3}\ln CURB_{i,t}+\beta_{4}lnLURB_{i,t}+\varepsilon_{i,t}\;$$

where *CE* is carbon emissions; *PURB*, *EURB*, *CURB*, and *LURB* are population urbanization, economic urbanization, consumption urbanization, and living urbanization, respectively, which are calculated using the method in Section 3.3.2; $\beta_{1}$, $\beta_{2}$, $\beta_{3}$, and $\beta_{4}$ are coefficients; the index *i* represents the province and *t* represents time; and $\varepsilon_{i,t}$ represents the error term. 

### 3.2. Spatial Econometrics Model

#### 3.2.1. Spatial Correlation

It is important to test the spatial correlation before establishing a spatial econometric model. There are numerous methods to test and measure spatial correlation, in which Moran’s I index is the most common method [47,48]. If Moran’s I index is not significantly zero, there is spatial correlation between variables. Moran’s I is an indicator whose value is normalized to [−1,1] after variance normalization. Moran’s I > 0 indicates a positive spatial correlation. Moran’s I < 0 indicates a negative spatial correlation. Moran’s I can be used to find out whether there exist spatial agglomerations of the variables. Moran’s I index is measured as below:

(2) $$Moran^{\prime }s\;I=\left(\sum_{i=1}^{n}\sum_{j=1}^{n}W_{ij}\left(Y_{i}-\bar{Y}\right)\left(Y_{j}-\bar{Y}\right)\right)/\left(S^{2}\sum_{i=1}^{n}\sum_{j=1}^{n}W_{ij}\right)\;S^{2}=\frac{1}{n}\sum_{i=1}^{n}{\left(Y_{i}-\bar{Y}\right)}^{2}\;\bar{Y}=\sum_{i=1}^{n}Y_{i}\;W_{ij}=\{\begin{array}{cc} 1, & i\neq j\\ 0, & i=j \end{array}$$

where $Y_{i}$ and $Y_{j}$ are carbon emissions in regions *i* and *j*; *W_ij_* is the spatial weight matrix; and *n* is the total number of study units. 

#### 3.2.2. Spatial Lag Model, Spatial Error Model, and Spatial Durbin Model

Furthermore, this paper analyzes the impact of multinational urbanizations on carbon emissions through the spatial econometrics model. Spatial econometric models have three forms, including the spatial lag model, the spatial error model, and the spatial Durbin model [49]. In Section 3.2.2, we introduce each spatial econometric model separately.

The spatial lag model (SLM) in this paper is defined as:

(3) $$lnCE_{i,t}=c+\rho \sum_{j=1}^{N}W_{i,j}lnCE_{j,t}+\beta_{1}lnPURB_{i,t}+\beta_{2}lnEURB_{i,t}+\beta_{3}lnCURB_{i,t}+\beta_{4}lnLURB_{i,t}+\varepsilon_{i,t}$$

where $lnCE_{i,t}$ and $lnCE_{j,t}\;$ denote carbon emissions of region *i* and region *j* at time *t*; ρ is the spatial correlation between $lnCE_{i,t}$ and $lnCE_{j,t};$ *W_ij_* is the spatial weight matrix; $lnPURB_{i,t}$, $lnEURB_{i,t}$, $lnCURB_{i,t}$, and $lnLURB_{i,t}$ are independent variables of region *i* at time *t*; c is the constant term; and $\beta_{1}$, $\beta_{2}$, $\beta_{3}$, and $\beta_{4}$ are coefficients to be confirmed.

The spatial error model (SEM) in this paper is formulated as:

(4) $$lnCE_{i,t}=c+\beta_{1}lnPURB_{i,t}+\beta_{2}lnEURB_{i,t}+\beta_{3}lnCURB_{i,t}+\beta_{4}lnLURB_{i,t}+\mu_{i,t}\;\mu_{i,t}=\gamma \sum_{j=1}^{N}W_{i,j}\mu_{i,t}+\varepsilon_{i,t}$$

where $lnCE_{i,t}$, $lnPURB_{i,t}$, $lnEURB_{i,t}$, $lnCURB_{i,t}$, $lnLURB_{i,t}$ c, $\beta_{1}$, $\beta_{2}$, $\beta_{3}$, and $\beta_{4}$ are defined as in Formula (3); $\mu_{i,t}$ denotes spatial error auto-correlation; γ is the spatial auto-correlation index.

**Figure 1:**
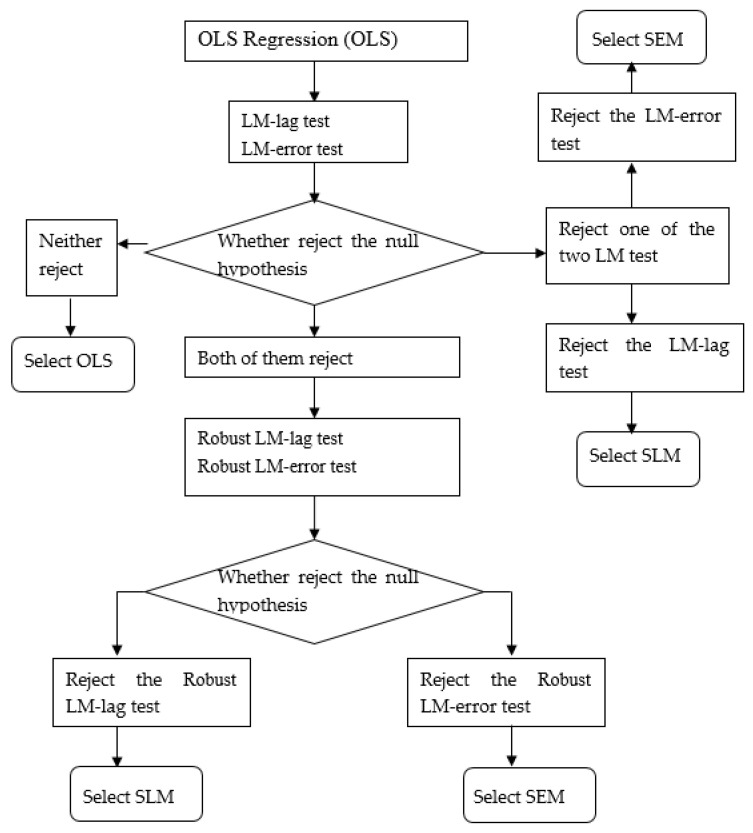
The selection of the SLM and SEM.

The selection processes of SLM and SEM are as follows: (1) We conduct the Lagrange Multiplier lag test (LM-lag test) and Lagrange Multiplier error test (LM-error test). (2) If neither the null hypothesis of the LM-lag test or the LM-error test is rejected, we select the ordinary least square (OLS) regression model. (3) If only the null hypothesis of the LM-lag test is rejected, we select SLM. If only the null hypothesis of the LM-error test is rejected, we select SEM. (4) If both the null hypothesis of the LM-lag test and the LM-error test are rejected, we conduct a robust LM-lag test and robust LM-error test. If only the null hypothesis of the robust LM-lag test is rejected, we select SLM. If only the null hypothesis of the robust LM-error test is rejected, we select SEM. The selection process of the SLM and SEM is shown in Figure 1.

**Figure 2:**
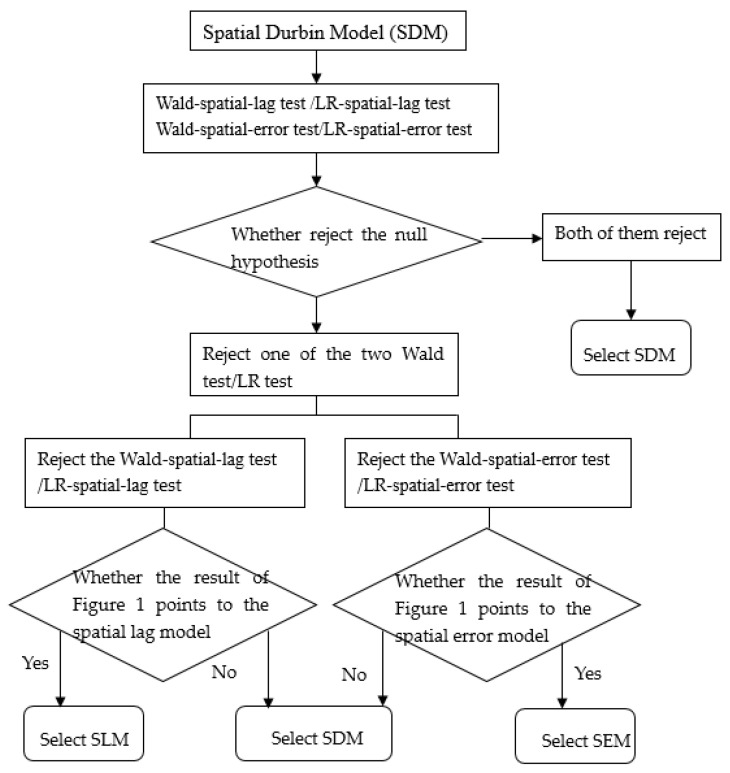
The selection of the SLM, SEM, and SDM.

The spatial Durbin model (SDM) (Formula (5)) extends the SLM with spatially lagged independent variables [50]. The selection processes of SLM, SEM, and SDM are as follows: (1) We conduct the Wald-spatial-lag test (or LR-spatial-lag test) and Wald-spatial-error test (or LR-spatial-error test). (2) If both the null hypothesis of the Wald-spatial-lag test and the Wald-spatial-error test are rejected, we select SDM. (3) If only the null hypothesis of the Wald-spatial-lag test (or LR-spatial-lag test) is rejected, we select SLM (if we select SLM in Figure 1) or SDM (if we do not select SLM in Figure 1). (4) If only the Wald-spatial-error test (or LR-spatial-error test) is rejected, we select SEM (if we select SEM in Figure 1) or SDM (if we do not select SEM in Figure 1). The selection process of the SLM, SEM, and SDM is shown below (Figure 2).

(5) $$\begin{array}{c} lnCE_{i,t}=c+\rho \sum_{j=1}^{N}W_{i,j}lnCE_{j,t}+\beta_{1}lnPURB_{i,t}+\beta_{2}lnEURB_{i,t}+\beta_{3}lnCURB_{i,t}+\beta_{4}lnLURB_{i,t}\\ \;\;\;\;\;\;+\theta_{1}\sum_{j=1}^{N}W_{i,j}\beta_{1}lnPURB_{i,j,t}+\theta_{2}\sum_{j=1}^{N}W_{i,j}\beta_{2}lnEURB_{i,j,t}+\theta_{3}\sum_{j=1}^{N}W_{i,j}\beta_{3}lnSURB_{i,j,t}\\ \;\;\;\;\;\;+\theta_{4}\sum_{j=1}^{N}W_{i,j}\beta_{4}lnLURB_{i,j,t}+\varepsilon_{i,t} \end{array}$$

where $lnCE_{i,t}$, $lnPURB_{i,t}$, $lnEURB_{i,t}$, $lnCURB_{i,t}$, $lnLURB_{i,t}$, c, $\rho ,\;\beta_{1}$, $\beta_{2}$, $\beta_{3}$, and $\beta_{4}$ are regulated the same as in Formulas (3) and (4). θ is a vector of coefficients to be confirmed. 

### 3.3. Variable Definitions

#### 3.3.1. Calculation of Carbon Emissions

We calculate carbon emission according to the 2006 IPCC report. The formula is as below:

(6) $$CE=\sum_{j=1}^{n}Energy_{j}\times Coefficient_{j}$$

where *j* denotes the type of energy, $Energy_{j}$ shows the expenditure of the *j*th energy, $Coefficient_{j}$ shows the Carbon emissions coefficient of the *j*th energy, and the data are obtained from the IPCC. Taking the eight major fossil fuels, including coal, coke, gasoline, kerosene, diesel, fuel oil, liquefied petroleum gas, and natural gas, into consideration, this paper calculates the carbon emissions of 30 provinces in China (except for Hong Kong, Macau, Taiwan, and Tibet due to the availability of the data) from 1997 to 2015.

#### 3.3.2. Four-Dimensional Urbanizations

Urbanization includes not only population transfer, but also various aspects such as economic structure, resident lifestyle, and urban land development. This paper builds an urbanization evaluation system from four dimensions [23,51], (Table 1).

We use the entropy weight method [52] to calculate the weight of variables, which is an objective weighting method. 

Firstly, the standardized value of the indicator $x_{j}\;$is calculated as follow:

(7) $$Y_{ij}=X_{ij}/\sum_{i=1}^{m}X_{ij}\;(i=1,\;2,\ldots ,\;m;\;j=1,\;2,\ldots ,\;12)$$

where $X_{ij}$ is the value of indicator *j* in year *i*; m is the number of years.

Secondly, the entropy value of the indicator $x_{j}$is calculated as follow:

(8) $$E_{j}=-\frac{1}{lnm}\sum_{i=1}^{m}Y_{ij}\times lnY_{ij}$$

Thirdly, the weight of the indicator $x_{j}$ is calculated as follows:

(9) $$\begin{array}{c} w_{j}=\left(1-E_{j}\right)/\sum_{j=1}^{3}\left(1-E_{j}\right)\;\mathrm{for}\;j=1,\;2,\;3\\ w_{j}=\left(1-E_{j}\right)/\sum_{j=4}^{6}\left(1-E_{j}\right)\;\mathrm{for}\;j=4,\;5,\;6\\ w_{j}=\left(1-E_{j}\right)/\sum_{j=7}^{9}\left(1-E_{j}\right)\;\mathrm{for}\;j=7,\;8,\;9\\ w_{j}=\left(1-E_{j}\right)/\sum_{j=10}^{12}\left(1-E_{j}\right)\;\mathrm{for}\;j=10,\;11,\;12 \end{array}$$

Finally, the value of population urbanization, economic urbanization, consumption urbanization, and living urbanization in year *i* is calculated as Formula (10) to (13), respectively.

(10) $${\mathrm{PURB}}_{i}=\sum_{j=1}^{3}w_{j}\times X_{ij}$$

(11) $${\mathrm{EURB}}_{i}=\sum_{j=4}^{6}w_{j}\times X_{ij}\;$$

(12) $${\mathrm{CURB}}_{i}=\sum_{j=7}^{9}w_{j}\times X_{ij}\;$$

(13) $${\mathrm{LURB}}_{i}=\sum_{j=10}^{12}w_{j}\times X_{ij}\;$$

### 3.4. Data Source

This paper utilizes panel data of 30 provinces in China (excluding Hong Kong, Macau, Taiwan, and Tibet due to the availability of the data) from 1997 to 2015 for empirical analysis. The original data for calculating carbon emissions chooses from the China Energy Statistics Yearbook. The original data for calculating four-dimensional urbanizations comes from the China Statistical Yearbook and the statistical database of China’s economic and social development.

## 4. Empirical Results and Discussions

### 4.1. Descriptive Statistics

The descriptive statistics for the main variables are shown in Table 2. It shows that carbon emissions in China have a standard deviation of 0.8212, with a minimum value of 6.1774, a maximum value of 10.8188. It indicates that regional differences in carbon emission cannot be ignored. Compared to population, economic, and living urbanization, consumption urbanization has a large variation, with a standard deviation of 0.7657, and a mean value of 1.3636. This may be due to the following: (1) Different regions of China are at diverse phases of economy growth. So, there are great differences in residents’ disposable income in China. (2) People’s living habits and consumption preferences are quite different. In addition, we see that the statistics of the variables of different regions differ greatly. There are great regional differences of carbon emissions and four-dimensional urbanizations in China.

**Figure 3:**
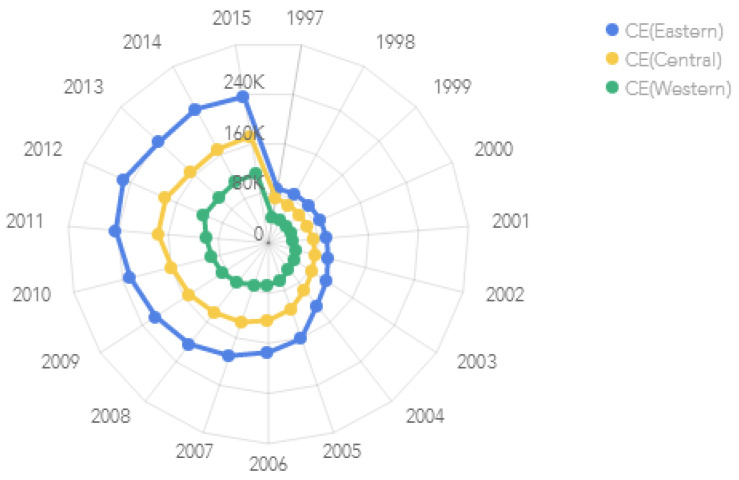
Carbon emissions of eastern, central, and western China from 1997 to 2015 (Unit: 10,000 tons).

There is obvious regional diversity of carbon emissions in China. Eastern China has the largest carbon emissions, followed by central China and western China (Figure 3). 

**Figure 4:**
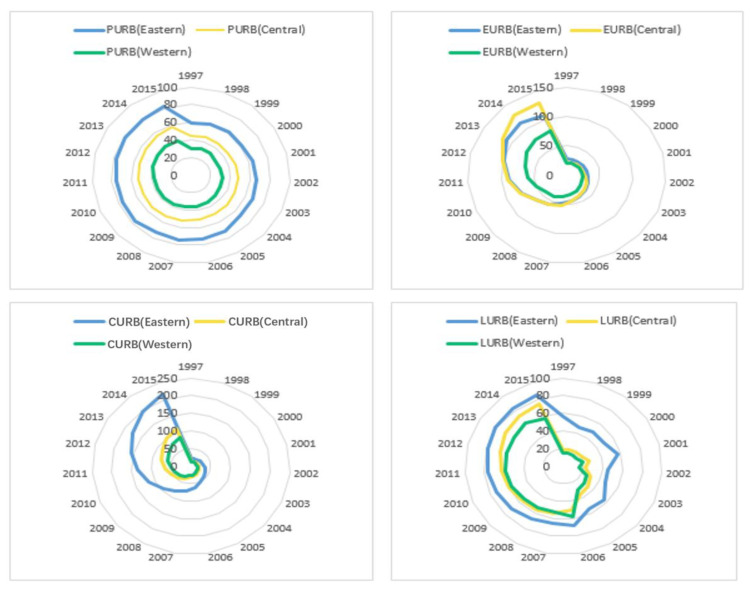
Four-dimensional urbanizations of eastern, central, and western China from 1997 to 2015 (Unit: %).

Four dimensional urbanizations of eastern, central, and western China from 1997 to 2015 are shown below (Figure 4). Population urbanization had significant regional differences in three regions of China. Eastern China has the largest population urbanization, followed by central China and western China. From 1997 to 2015, population urbanization increased steadily by about 20% in all regions. From 1997 to 2001, economic urbanization in all regions was roughly the same, with slow growth. From 2001 to 2011, the growth rate of economic urbanization in the western China slowed down and was less than economic urbanization in the central China and eastern China. Since 2011, economic urbanization in central China has developed rapidly. Eastern China has the largest consumption urbanization, followed by central China and western China. Over time, regional differences have widened. Consumption urbanization in eastern China is almost four times that of the western region. In terms of living urbanization, it shows an upward trend in all three regions. Eastern China has the largest living urbanization, followed by central China and western China from 1997 to 2015. In eastern China, durable consumer goods are basically popular. High-tech industry and the tertiary industry are developing rapidly in eastern China. In western China, the reform and opening up has gradually deepened and expanded. The industrial structure in the western China is updating, but there is still a large gap compared with eastern China. Insufficient funds and backward technology are the main bottlenecks which restrict the industrial structure updating of western China.

### 4.2. The Results of Panel Data Model

Before we do the regression of panel data model, we perform multiple tests to make our results more robust. Firstly, in order to evaluate whether the variables are stationary, we perform panel unit root test. Secondly, it is necessary to make sure whether there is a long-term relationship between variables. Therefore, we do the panel integration test to make sure that there is a long-term cointegration relationship between four-dimensional urbanizations and carbon emissions. Cointegration analysis is a prerequisite for estimating long-term regression coefficients. Multicollinearity means that the independent variables in the model are correlated [53]. Thirdly, we use the method of the variance inflation factor (VIF) to examine whether there exists multicollinearity between the independent variables. Fourthly, we use the Modified Wald test, Sargan–Hansen test, and correlation test to test for heteroskedasticity of the variables. Finally, we use a panel data model for regression. We estimate panel data models at the national and regional levels to analyze the impact of four-dimensional urbanizations on carbon emissions.

#### 4.2.1. Panel Unit Root Test

This paper chooses the Phillips and Perron test (PP test) and Levin–Lin–Chu test (LLC test) to verify the stationarity of the variables [54,55]. The outcomes of whole panel data and the three regions in China are presented below (Table 3 and Table 4). The outcomes show that carbon emission and four-dimensional urbanizations of all panel data are first-order stationary.

#### 4.2.2. Panel Cointegration Test

Kao test [56] results are shown below (Table 5). It means that there is a long-term cointegration relationship between four-dimensional urbanizations and carbon emission. 

#### 4.2.3. Multicollinearity Test

Multicollinearity means that the independent variables in the model are correlated [53]. The OLS estimator is ineffective in the case of multicollinearity. We use the method of variance inflation factor (VIF) to examine the presence of multicollinearity. When VIF > 10, there exists multicollinearity between independent variables [57,58]. If the VIF values of four-dimensional urbanizations are less than 10, there is no multicollinearity of four-dimensional urbanizations (Table 6).

#### 4.2.4. Modified Wald Test, Sargan–Hansen Test, and Correlation Test

The modified Wald test is used to test the heteroskedasticity [59]. According to the results, there is heteroscedasticity in all four regions (Table 7).

Due to the heteroscedasticity, the Sargan–Hansen test [60] is utilized to identify whether to select the fixed effect model or the random effect model. From Table 8, we see that the fixed effect (FE) model is proper for the panel data of whole regions, eastern China, and central China. The random effect (RE) model is proper for the panel data of western China.

Furthermore, we use the Wooldridge test [59] and Pearson test to test the autocorrelation and cross-sectional correlation. The outcomes indicate that autocorrelation and cross-sectional correlation exist in all the panel data (Table 9).

#### 4.2.5. Panel Regression Results

Because autocorrelation and cross-sectional correlation exist in all panel data, the estimation outcomes of FE models are biased. We choose the regression with cluster robustness standard errors (cluster), the feasible generalized least squares (FGLS), and the regression with Driscolle and Kraay standard errors (DK) to estimate the models [61]. The outcomes are listed below (Table 10, Table 11, Table 12 and Table 13).

Because there exists autocorrelation, heteroskedasticity, and cross-sectional correlation of whole regions, eastern and central China, FGLS and DK are reliable. DK is more reliable than FGLS for panel data of whole regions, if the number of years is less than that of cross-sections (Table 10, column 4). FGLS is the most reliable estimation method, if the number of cross-sections is less than that of years. The results of the FGLS model are the most reliable for panel data of eastern and central China (Table 11, column 7 and Table 12, column 11). When there exist heteroskedasticity and autocorrelation, the ordinary least square regression model with cluster robustness standard errors is applied more often [59]. As a result, the estimation results of the RE_cluster model is the most reliable for panel data of western China (Table 13, column 14).

From the results, we see that population urbanization has a positive effect on carbon emissions of the whole regions, eastern China, and western China. An increase of 1% in population urbanization leads to an increase of 0.6357%, 0.5087%, and 0.8094% in carbon emissions of the whole regions, eastern China, and western China, respectively. The elasticity of population urbanization in central China is not statistically significant. As China’s urbanization progresses, more and more people come to urban areas. The increase in population leads to more congested traffic, severe housing problems, and more energy consumption. Talents bring technological advantages to cities, but the proportion of talents in China is relatively low at present. The benefits brought by talents in urban areas are far less than the negative effects of large numbers of people entering cities. As a result, the environment bears more pressure and carbon emissions increase.

The elasticity of economic urbanization is 0.1633, 0.126, 0.0647 for the whole regions, central China, and eastern China, respectively. The elasticity of economic urbanization in western China is not statistically significant. Economic urbanization promotes carbon emissions of the whole regions, central China, and eastern China. Since joining the WTO in 2001, China’s industrial structure has been continuously optimized. China’s industrial restructuring is to develop advanced manufacturing, increase the proportion and level of the service industry, strengthen infrastructure construction, and optimize the industrial structure of urban and rural areas. However, there are still many problems in China’s economic development. Economic development and carbon emissions have not yet been decoupled [60]. Economic urbanization is accompanied by more carbon emissions in most areas. China’s current industrial technology is relatively low. The industry concentration is not high, so the effective concentration of capital and brands cannot be achieved in the market competition. The industrial structure of eastern China, central China, and western China is similar. To optimize the industrial structure and accelerate the realization of the goal of industrial upgrading, China needs to solve various problems existing in the industrial structure.

The elasticity of consumption urbanization is 0.2558, 0.3244, 0.2272, 0.3456 for the whole regions, eastern China, central China, and western China, respectively. Consumption urbanization promotes carbon emissions of the whole regions, eastern China, central China, and western China. Carbon emissions caused by household consumption includes not only the carbon emissions generated by the direct consumption of energy such as daily cooking, heating, and travel, but also the indirect carbon emissions generated by the production and transportation of personal consumer products and services. At present, people’s concept of low-carbon consumption is not mature. With the improvement of people’s living standards, most of the consumer goods and services purchased are environmentally unfriendly products; especially, the number of private cars in cities is increasing. Energy consumption for living and transportation continues to increase. The development of consumption urbanization promotes carbon emissions.

The elasticity of living urbanization is 0.1441, 0.0795, 0.1129 for the whole regions, eastern China, and central China, respectively. Living urbanization has a positive impact on carbon emissions for the whole regions, eastern China, and central China. With the development of urbanization, resources are more intensive, urban infrastructure is continuously improved, and public transportation is more developed. The agglomeration of urban public facilities achieves economic scale. The compact of urban space also reduces the travel distance of residents and reduces energy consumption. However, excessive urban density causes traffic congestion and releases more carbon dioxide. The proportion of private cars in cities is too high, and people’s habit of taking public transportation is still developing. At the current stage in China, living urbanization has a greater negative effect on carbon emission reduction.

As a large country with a vast territory, China cannot be synchronized in the development of various regions. The differences in the empirical results indicate that economic development, living habits, and social features in different regions of China are different.

### 4.3. The Results of Spatial Econometrics Model

#### 4.3.1. Moran’s I of Carbon Emissions

We use Formula (2) to calculate the Moran’s I of carbon emissions from 1997 to 2015 in China. From Table 14, we see that all the Moran’s I of carbon emissions from 1997 to 2015 in China are larger than 0. Most of them are significant at the 5% level, while the Moran’s I in 2003, 2012, and 2015 are significant at the 10% level. The results indicate that carbon emissions in China present a significant spatial aggregation. It is important to use spatial econometrics model to study the impacts of four-dimensional urbanizations on carbon emissions.

Then, we use the software GeoDa to visualize and analyze the Moran’s I of carbon emissions from 1997 to 2015. GeoDa is a software program that allows users to visualize and analyze Moran’s I [61]. There are four types of spatial agglomerations that are widely used [62]: (1) H-H agglomeration: High carbon emissions regions are adjacent to other high carbon emissions regions; (2) L-L agglomeration: Low carbon emissions regions are adjacent to other low carbon emissions regions; (3) L-H agglomeration: Low carbon emissions areas are adjacent to high carbon emissions regions; (4) H-L agglomeration: High carbon emissions areas are adjacent to low carbon emissions regions. Table 15 shows the spatial agglomerations of China’s 30 provinces in 1997, 2000, 2005, 2010, and 2015.

By analyzing all provinces of China in these five years, we draw the following findings: (1) There are six provinces always in H-H agglomeration in these five years, including Shandong, Henan, Shanxi, Liaoning, Jiangsu, and Hebei. (2) There are seven provinces always in L-H agglomeration in these five years, including Beijing, Tianjin, Jilin, Chongqing, Shaanxi, Jiangxi, Guangxi. (3) There are four provinces always in L-L agglomeration in these five years, including Xinjiang, Gansu, Ningxia, Qinghai. (4) There are two provinces always in H-L agglomeration in these five years, including Guangdong and Sichuan. (5) Inner Mongolia was in H-H agglomeration in 2005, 2010, and 2015, and was in L-H agglomeration in 1997 and 2000. Shanghai was in L-H agglomeration in 1997, 2005, 2010, 2015, and was in H-H agglomeration only in 2000. Hunan was in H-L agglomeration in 1997, 2005, 2010, and 2015, and was L-H agglomeration only in 2000. 

Generally, about 25% of the provinces were in H-H agglomeration in these five years. About 33% of the provinces were in L-H agglomeration in these five years. About 22% of the provinces were in L-L agglomeration in these five years. About 13% of the provinces were in H-L agglomeration in these five years. The total number of provinces in H-H agglomeration and L-L agglomeration was equal to that of H-L agglomeration and L-H agglomeration in 1997, 2000, and 2005. The total number of provinces in H-H agglomeration and L-L agglomeration was slightly larger than that of H-L agglomeration and L-H agglomeration in 2010 and 2015. 

#### 4.3.2. Regression Results of Spatial Econometrics Model

Firstly, referring to the selection process shown in Figure 1, we conduct LM tests. The LM test results of the model with spatial and time-period fixed effects support the SEM. In addition, the LR test results (listed in the last two rows) indicate that the SEM with spatial and time-period fixed effects is the most suitable model (Table 16).

Secondly, referring to the selection process shown in Figure 2, we conduct Wald and LR tests. Table 16 gives the outcomes of the Wald and LR tests. The outcomes of the Wald test and the LR tests indicate that the SDM is the most suitable model. 

The Hausman’s specification test is utilized to examine the random effects model against the fixed effects model [59]. The outcomes (130.4353, 9, df, *p* < 0.01) show that the fixed effect model is acceptable (Table 16). According to the results of SDM with a fixed effect shown in column 2 in Table 17, the regression coefficients of population urbanization, economic urbanization, and consumption urbanization are all significantly positive. It means that population urbanization, economic urbanization, and consumption urbanization promote carbon emissions. Chinese government should balance the development of urbanization and the decrease of carbon emissions.

The SDM model is insufficient to explain the marginal effects of the independent variable on the dependent variable [49]. Consequently, the direct, indirect, and total effects are further estimated into the modeling process (Table 18). Using Stata to estimate the SDM model, it gives the result of direct, indirect, and total effects directly. 

In the direct effects, an increase of 1% in PURB, EURB, and CURB lead to 0.4223%, 0.2017%, and 0.3526% growth of CO_2_ emissions inside a same province, respectively (column 2, Table 18). The direct effect of the LURB is positive but not significant, proving that living urbanization has no significant influence on carbon emissions inside a same province. The indirect effects of EURB and CURB are significantly negative. The indirect effects of LURB are significantly positive. Specifically, an increase of 1% in EURB and CURB cause a 0.2973% and 0.2970% decrease of CO_2_ emissions in the neighboring provinces, respectively. An increase of 1% in LURB in the region causes a 0.1785% increase of CO_2_ emissions in the neighboring provinces. The indirect effect of PURB is insignificant. 

By decomposing the impact of urbanization on carbon emissions into direct effects and indirect effects, we analyze the effects of multi-dimensional urbanizations on carbon emissions comprehensively. Population urbanization has a positive effect on local carbon emissions and no significant effect on its neighboring provinces’ carbon emissions. 

Economic urbanization and consumption urbanization promote local carbon emissions and reduce carbon emissions in its neighboring provinces. However, the total effects of economic urbanization and consumption urbanization are not significant. The economic development and the improvement of the consumption level of a certain region often attract more people to work and live there. More people cause more daily consumption, including housing and transportation. As a result, more people coming from other region increase local carbon emissions and reduce the carbon emissions of neighboring regions.

Living urbanization has positive effects on local carbon emissions and its neighboring provinces’ carbon emissions. The total effect of living urbanization on local carbon emissions is also significantly positive. The increase of living urbanization results in more urban built-up area and more road construction. However, China’s current land use patterns and spatial pattern planning are not perfect. It is difficult to achieve economic scale. As a result, living urbanization increases local carbon emissions and carbon emissions of neighboring regions.

## 5. Policy Recommendations

### 5.1. Keep a Watchful Eye on the Carbon Emission Reduction Policies in the Neighboring Areas

Carbon emissions from an area will not only be impacted by the urbanization factors in a particular area itself but will also be impacted by urbanization and carbon emissions in its neighboring areas. When it comes to carbon emission decreasing policies, it is important to keep a watchful eye on the carbon emission decreasing policies and urbanization factors in the neighboring areas. To meet the target of carbon emission decreasing, regional information sharing mechanisms can be established in various regions, or carbon trading can be carried out.

### 5.2. Control Population Growth Reasonably and Promote a Rational Population Distribution

Controlling population growth reasonably is one of the necessary ways to decrease carbon emissions in course of urbanization. Facing the current policy of opening the second child, the relevant government departments should make efforts to control the population to avoid negative effects on the ecology. For any economy, it is difficult to achieve equilibrium in the period of rapid urbanization, and the same is true for China’s current urbanization. Nowadays, China’s population is unevenly distributed. The population imbalance between the east and the west is not conducive to the coordinated development between regions. So, it is necessary to promote a more rational population distribution. To achieve the goal, the population structure needs be optimized and the quality of the population should be improved. 

### 5.3. Speed Up Industrial Restructure and Develop Low-Carbon Industries

In the aspect of economic urbanization, it is important to speed up industrial restructure and actively guide the transformation of the national economy from a secondary industry to the tertiary industry, and to moderately increase energy efficiency and reduce carbon emissions.

Specifically, we can cultivate and develop distinctive urban industrial systems based on urban environmental carrying capacity, factor endowments, and comparative advantages first; second, transform and upgrade traditional industries, and expand emerging industries such as environmentally friendly industry, biology industry, new energy industry, and new materials industry; third, adapt to the requirements of transformation and the upgrade of manufacturing industries, promote the professionalization and marketization of productive service industries, guide the gathering of the productive service industry in central cities and densely-manufactured regions; and last, in order to adapt to the diversification of consumer demand, it is meaningful to expand service provision and perfect service quality; make efforts to promote the formation of a service economy-based industrial structure in megacities and big cities.

### 5.4. Strengthen People’s Awareness of Environmental Protection

In the aspect of consumption urbanization, it is essential to strengthen the promotion of low-carbon life and energy-saving reduction from the cooperated efforts from the government, society, and residents. Firstly, the government can encourage the development of the low-carbon economy through system design and policies, such as increasing support for public transportation and giving certain tax preferences to environmental protection industries. Secondly, social media can raise people’s awareness of environmental protection through advertisements and lectures. For example, lectures on garbage classification in the community can promote residents’ awareness of resource recycling. Thirdly, the increase in per capita disposable income will increase consumer demand for transportation and household appliances, and changes in consumption structure will increase energy expenditure and carbon emissions. So, residents need to pay attention to environmentally friendly factors when they consume, such as purchasing more energy-saving appliances.

### 5.5. Use Land Reasonably and Improve Inter-Regional Urban Development Mechanism

From the perspective of land use and urban planning, urban planning must follow the concept of being green and low-carbon. It should be transformed from expansionary planning into a plan that defines urban boundaries and optimizes spatial structure. For example, for land use for different purposes, detailed land rate and greening rate requirements should be planned scientifically to avoid overexpansion of land. Considering that the living urbanization of China has an obvious spatial spillover effect on carbon emissions, all the regions should use urban agglomerations as a platform to achieve the goal of establishing and improving inter-regional urban development mechanisms. For example, it is important to encourage low-carbon travel among residents and promote the establishment and sharing of infrastructure and public service facilities.

## 6. Conclusions

Using the panel data of 30 provinces in China from 1997 to 2015, this paper studies the impacts of urbanization on carbon emission. Regarding the impact of urbanization on carbon emissions, we study from the four dimensions of urbanization, including population urbanization, economic urbanization, consumption urbanization, and living urbanization. Moreover, we use the entropy weight method to measure the weight of each indicator to evaluate four-dimension urbanizations. In addition, we investigated the spatial correlation of carbon emissions, taking the spatial differences into consideration. The spatial Durbin model is finally selected to analyze the impacts of urbanizations on carbon emission. We make conclusions as follows:

Firstly, from the results of the panel data model, four dimensions of urbanization all play a significant role in promoting carbon emissions for the whole regions. Population urbanization has the greatest impact, while living urbanization has the least impact. In eastern China, four dimensions of urbanization all play a significant role in promoting carbon emissions, which is consistent with the conclusion of the whole regions. Population urbanization has the greatest impact, and economic urbanization has the least impact in eastern China. In central China, the effect of population urbanization on carbon emissions is not significant. The role of consumption urbanization is the largest, and the effects of economic urbanization and living urbanization are almost the same. In western China, the effects of economic urbanization and living urbanization on carbon emissions are not significant. The impact of population urbanization is greater than that of consumption urbanization.

Secondly, the Moran’s I of China’s carbon emissions from 1997 to 2015 were all greater than 0 significantly, which indicates that the spatial aggregation of carbon emissions of 30 provinces in China is significant. Spatial correlation should not be neglected when we study the influence of urbanization on carbon emissions. The total number of provinces in H-H agglomeration and L-L agglomeration was equal to that of H-L agglomeration and L-H agglomeration in 1997, 2000, and 2005. The total number of provinces in H-H agglomeration and L-L agglomeration was slightly larger than that of H-L agglomeration and L-H agglomeration in 2010 and 2015.

Thirdly, from the results of the spatial econometrics model, population urbanization has a positive effect on local carbon emissions and no significant effect on its neighboring provinces’ carbon emissions. Economic urbanization and consumption urbanization promote local carbon emissions and reduce carbon emissions in its neighboring provinces. Living urbanization has positive effects on local carbon emissions and its neighboring provinces’ carbon emissions. 

This paper proposes some recommendations for the carbon emission decreasing during urbanization. First, establishment and improvement of coordination mechanisms and information sharing mechanisms across regions should also be considered. Second, control population growth reasonably and optimize population structure in order to achieve an orderly flow and rational distribution of the population. Third, the assessment mechanism of the local government should include not only economic indicators but also other indicators.

## Figures and Tables

**Table 1:** Urbanization Variables.

Dimensions	Symbol	Variable	Unit
Population urbanization	PURB	The proportion of urban population (*X*_1_)	%
		Urban population size (*X*_2_)	10 thousand
		The proportion of aging population (*X*_3_)	%
Economic urbanization	EURB	Percentage of secondary industry in GDP (*X*_4_)	%
		Real estate investment completed (*X*_5_)	10^8^ RMB
		energy efficiency (*X*_6_)	10^4^ Yuan/10^3^ kgTce
Consumption urbanization	CURB	Urban residents’ disposable income (*X*_7_)	Yuan
		public transport vehicles owned by per 10,000 people (*X*_8_)	
		Private cars owned by per 10,000 people (*X*_9_)	
Living urbanization	LURB	Urban population density (*X*_10_)	Urban population per km^2^
		Road area per capita (*X*_11_)	m^2^
		Urban built-up area (*X*_12_)	km^2^

**Table 2:** Statistics description of carbon emission and four-dimension urbanizations.

	Variables	Mean	Std. Dev	Min	Max	Observations
Whole regions	lnCE	9.1004	0.8212	6.1774	10.8188	570
	lnPURB	1.5394	0.5159	−0.5872	2.3952	570
	lnEURB	1.4196	0.6422	−0.0699	3.2442	570
	lnCURB	1.3636	0.7657	−0.1773	3.4168	570
	lnLURB	1.4569	0.5661	−0.2957	2.7308	570
Eastern China	lnCE	9.1843	0.9246	6.2482	10.8188	228
	lnPURB	1.6840	0.4638	0.0316	2.3952	228
	lnEURB	1.3504	0.6312	−0.0699	2.9903	228
	lnCURB	1.5918	0.8298	−0.1773	3.4168	228
	lnLURB	1.6031	0.3748	0.4523	2.7308	228
Central China	lnCE	9.3663	0.5333	8.1291	10.3056	171
	lnPURB	1.6772	0.3057	0.9752	2.2744	171
	lnEURB	1.6328	0.6709	0.6065	3.2038	171
	lnCURB	1.3045	0.6926	0.2623	3.0034	171
	lnLURB	1.4465	0.5869	0.1565	2.3069	171
Western China	lnCE	8.7226	0.7790	6.1774	10.1505	171
	lnPURB	1.2088	0.5941	−0.5872	2.2514	171
	lnEURB	1.2986	0.5759	−0.5872	2.2514	171
	lnCURB	1.1184	0.6555	0.1136	2.8401	171
	lnLURB	1.2724	0.6911	−0.2957	2.3467	171

**Table 3:** Unit root test results of whole panel data.

Unit Root Test	Variables	Fish-PP	LLC
Level	lnCE	15.8061	−2.53078 ***
	lnPURB	68.6858	−1.94476 **
	lnEURB	3.93320	−1.98812 **
	lnCURB	4.73390	2.56844
	lnLURB	53.3643	−1.63801 *
1st difference	lnCE	241.043 ***	−6.74072 ***
	lnPURB	672.889 ***	−13.4772 ***
	lnEURB	117.939 ***	−2.86401 ***
	lnCURB	200.690 ***	−5.86105 ***
	lnLURB	1391.53 ***	−8.59261 ***

Note: ***, **, and * denote statistical significance at 1%, 5%, and 10%, respectively.

**Table 4:** Unit root test results of panel data of eastern, central, and western China.

		Eastern China		Central China		Western China	
	Variables	Fish-PP	LLC	Fish-PP	LLC	Fish-PP	LLC
Level	lnCE	7.847	−2.552 ***	3.312	−1.270	4.647	−0.192
	lnPURB	29.949	−1.8384 **	3.474	−0.044	35.263 ***	−1.4140 *
	lnEURB	2.142	−1.966 **	0.371	−0.486	1.420	−0.961
	lnCURB	4.725	−1.528 *	0.004	2.596	0.005	5.947
	lnLURB	35.992 *	0.631	5.256	−1.033	12.1166	−2.319 **
1^st^ difference	lnCE	79.837 ***	−5.033 ***	52.868 ***	−3.774 ***	108.339 ***	−5.147 ***
	lnPURB	329.324 ***	−6.414 ***	191.158 ***	−6.567 ***	152.406 ***	−7.364 ***
	lnEURB	42.532 ***	−3.578 ***	40.9471 ***	−2.087 **	43.404 ***	−3.892 ***
	lnCURB	101.308 ***	−4.742 ***	28.2175 *	−2.381 ***	71.165 ***	−3.334 ***
	lnLURB	909.478 ***	−4.916 ***	357.151 ***	−5.886 ***	124.902 ***	−4.141 ***

Note: ***, **, and * denote statistical significance at 1%, 5%, and 10%, respectively.

**Table 5:** Kao test results of panel data.

	ADF
Whole regions	−5.805119 ***
Eastern China	−3.785178 ***
Central China	−5.341595 ***
Western China	−5.341595 ***

Note: *** denotes statistical significance at 1%.

**Table 6:** VIF values of independent variables of panel models.

Variables	Whole Regions	Eastern China	Central China	Western China
lnPURB	1.41	1.49	1.61	2.06
lnEURB	1.92	1.68	8.66	2.24
lnCURB	2.88	2.73	7.02	4.50
lnLURB	2.00	1.57	4.49	2.34

**Table 7:** Heteroscedasticity tests’ results.

Whole Regions	Eastern China	Central China	Western China
674.59 ***	151.74 ***	1558.47 ***	1558.47 ***

Note: *** denotes statistical significance at 1%.

**Table 8:** Sargan–Hansen test results.

Whole Regions	Eastern China	Central China	Western China
18.493 ***	39.342 ***	27.080 ***	4.258

Note: *** denotes statistical significance at 1%.

**Table 9:** Results of autocorrelation and cross-sectional correlation test.

	Whole Regions	Eastern China	Central China	Western China
Autocorrelation test	F(1,29) = 72.477 ***	F(1,11) = 111.804 ***	F(1,8) = 292.464 ***	F(1,8) = 16.764 ***
Cross-sectional correlation test	14.720 ***	9.026 ***	3.279 ***	0.582

Note: *** denotes statistical significance at 1%.

**Table 10:** Estimation results of whole regions’ panel data.

Variables	(1)	(2)	(3)	(4)
	FE	FE_Cluster	FGLS	DK_FE
cons	7.3314 *** (0.0922)	7.3314 *** (0.2114)	7.3961 *** (0.0466)	7.3314 *** (0.1048)
lnPURB	0.6357 *** (0.0680)	0.6357 *** (0.1551)	0.6919 *** (0.0339)	0.6357 *** (0.0959)
lnEURB	0.1633 *** (0.0368)	0.1633 *** (0.0797)	0.1219 *** (0.0194)	0.1633 *** (0.0526)
lnCURB	0.2558 *** (0.0283)	0.2558 *** (0.0659)	0.2497 *** (0.0231)	0.2558 *** (0.0300)
lnLURB	0.1441 *** (0.0220)	0.1441 *** (0.0528)	0.0419 *** (0.0119)	0.1441 *** (0.0336)
R-squared	0.8463	0.8463	NA	0.8463
observations	570	570	570	570

Note: *** denotes statistical significance at 1%.

**Table 11:** Estimation results of eastern China’s panel data.

Variables	(5)	(6)	(7)	(8)
	FE	FE_Cluster	FGLS	DK_FE
cons	7.4067 *** (0.1810)	7.4067 *** (0.4967)	7.5863 *** (0.1041)	7.4067 *** (0.1638)
LnPURB	0.4401 *** (0.1122)	0.4401 *** (0.3441)	0.5087 *** (0.0490)	0.4401 *** (0.1033)
LnEURB	0.0801 (0.0608)	0.0801 (0.1197)	0.0647 ** (0.0295)	0.0801 (0.0876)
LnCURB	0.3125 *** (0.0417)	0.3125 *** (0.0956)	0.3244 *** (0.0240)	0.3125 *** (0.0463)
LnLURB	0.2688 *** (0.0581)	0.2688 *** (0.1329)	0.0795 *** (0.0202)	0.2688 *** (0.0666)
R-squared	0.8473	0.8473	NA	0.8473
observations	228	228	228	228

Note: *** and ** denote statistical significance at 1% and 5% respectively.

**Table 12:** Estimation results of central China’s panel data.

Variables	(9)	(10)	(11)	(12)
	FE	FE_Cluster	FGLS	DK_FE
cons	8.0209 *** (0.3882)	8.0209 *** (0.9101)	8.3936 *** (0.2202)	8.0209 *** (0.2813)
LnPURB	0.2345 (0.2598)	0.2345 (0.5831)	0.1409 (0.1274)	0.2345 (0.2032)
LnEURB	0.3170 *** (0.1099)	0.3170 ** (0.1063)	0.1260 ** (0.0547)	0.3170 *** (0.1039)
LnCURB	0.0465 (0.0953)	0.0465 (0.1100)	0.2272 *** (0.0534)	0.0465 (0.0855)
LnLURB	0.2585 *** (0.0516)	0.2585 ** (0.1037)	0.1129 *** (0.0267)	0.2585 *** (0.0664)
R-squared	0.8466	0.8466	NA	0.8466
observations	171	171	171	171

Note: *** and ** denote statistical significance at 1% and 5% respectively.

**Table 13:** Estimation results of western China’s panel data.

Variables	(13)	(14)	(15)	(16)
	RE	RE_Cluster	FGLS	DK_RE
cons	7.1203 *** (0.1369)	7.1203 *** (0.2291)	7.1203 *** (0.1369)	7.1203 *** (0.1598)
LnPURB	0.8094 *** (0.0906)	0.8094 *** (0.1269)	0.8094 *** (0.0906)	0.8094 *** (0.1118)
LnEURB	0.1343 ** (0.0668)	0.1343 (0.1322)	0.1343 ** (0.0668)	0.1343 * (0.0730)
LnCURB	0.3456 *** (0.0550)	0.3456 *** (0.1033)	0.3456 *** (0.0550)	0.3456 *** (0.0623)
LnLURB	0.0495 (0.0344)	0.0495 (0.0634)	0.0495 (0.0344)	0.0495 (0.0417)
R-squared	0.8701	0.8701	NA	0.8701
observations	171	171	171	171

Note: ***, **, and * denote statistical significance at 1%, 5%, and 10%, respectively.

**Table 14:** Moran’s I of Carbon Emission from 1997 to 2015 in China.

Year	Moran’s I	*p*-Value
1997	0.1738 **	0.0440
1998	0.1873 **	0.0410
1999	0.2037 **	0.0320
2000	0.2138 **	0.0280
2001	0.2392 **	0.0200
2002	0.2189 **	0.0200
2003	0.1609 *	0.0700
2004	0.2094 **	0.0380
2005	0.2441 **	0.0180
2006	0.2547 **	0.0130
2007	0.2619 ***	0.0070
2008	0.2309 **	0.0230
2009	0.2100 **	0.0180
2010	0.2113 **	0.0230
2011	0.2175 **	0.0200
2012	0.1815 *	0.0520
2013	0.1815 **	0.0350
2014	0.1941 **	0.0310
2015	0.1657 *	0.0600

Note: ***, **, and * denote statistical significance at 1%, 5%, and 10%, respectively.

**Table 15:** The spatial agglomerations of China’s 30 provinces in 1997, 2000, 2005, 2010, and 2015.

Year	H-H Agglomeration	L-H Agglomeration	L-L Agglomeration	H-L Agglomeration	Others
1997	Shandong, Henan, Anhui, Shanxi, Liaoning, Jiangsu, Hebei, Hubei	Beijing, Tianjin, Jilin, Shanghai, Chongqing, Guangxi, Jiangxi, Shaanxi, Inner Mongolia, Yunnan	Xinjiang, Gansu, Ningxia, Qinghai, Fujian, Zhejiang	Guangdong, Sichuan, Hunan, Heilongjiang	Hainan, Guizhou,
2000	Shandong, Henan, Anhui, Shanxi, Liaoning, Jiangsu, Hebei, Shanghai	Beijing, Tianjin, Jilin, Chongqing, Shaanxi, Jiangxi, Guangxi, Fujian, Hunan, Inner Mongolia	Xinjiang, Gansu, Ningxia, Qinghai, Yunnan, Heilongjiang	Guangdong, Sichuan, Hubei, Guizhou	Hainan, Zhejiang
2005	Shandong, Henan, Shanxi, Liaoning, Jiangsu, Hebei, Inner Mongolia	Beijing, Tianjin, Jilin, Chongqing, Shaanxi, Anhui, Shanghai, Jiangxi, Guangxi, Fujian	Xinjiang, Gansu, Ningxia, Qinghai, Yunnan, Heilongjiang, Guizhou	Guangdong, Sichuan, Hunan, Zhejiang	Hainan, Hubei
2010	Shandong, Henan, Shanxi, Liaoning, Jiangsu, Hebei, Inner Mongolia	Beijing, Tianjin, Jilin, Chongqing, Shaanxi, Anhui, Shanghai, Jiangxi, Guangxi, Fujian, Heilongjiang	Xinjiang, Gansu, Ningxia, Qinghai, Yunnan, Guizhou, Zhejiang	Guangdong, Sichuan, Hunan, Hubei	Hainan
2015	Shandong, Henan, Shanxi, Liaoning, Jiangsu, Hebei, Inner Mongolia, Hubei	Beijing, Tianjin, Jilin, Shanghai, Chongqing, Guangxi, Fujian, Shaanxi, Jiangxi	Xinjiang, Gansu, Ningxia, Qinghai, Yunan, Guizhou, Zhejiang	Guangdong, Sichuan, Hunan,	Hainan, Heilongjiang, Anhui

**Table 16:** LM test results and LR test results.

	Pooled OLS	Spatial Fixed Effects	Time-Period Fixed Effects	Spatial and Time-Period Fixed Effects
Intercept	6.9369 ***			
LnPURB	1.0480 ***	0.6357 ***	1.0516 ***	0.4277 ***
LnEURB	0.1126 ***	0.1633 ***	0.1123 ***	0.1526 ***
LnCURB	0.3337 ***	0.2558 ***	0.3438 ***	0.1340 **
LnLURB	−0.0444	0.1441 ***	−0.1039 **	0.0443 *
LM-lag test	178.1159 ***	175.7228 ***	157.0918 ***	53.3272 ***
Robust LM-lag test	2.2518	10.8811 ***	1.6455	2.4368
LM-error test	304.0103 ***	167.5777 ***	272.8361 ***	58.9688 ***
Robust LM-error test	128.1461 ***	2.7360 *	117.3898 ***	8.0783 ***
R-squared	0.7648	0.8463	0.7095	0.0853
Adj R-squared	0.7631	0.8455	0.7079	0.0804
LR-test joint significance spatial fixed effects 1109.9212 ***, df = 30				
LR-test joint significance time-period fixed effects 130.9329 ***, df = 19				

Note: ***, **, and * denote statistical significance at 1%, 5%, and 10%, respectively.

**Table 17:** SDM with fixed effect and random Effect.

	SDM_FE	SDM_RE
Variable	Coefficient	Coefficient
LnPURB	0.4101 *** (4.1264)	0.489762 *** (4.6193)
LnEURB	0.2270 *** (6.3773)	0.215056 *** (5.2371)
LnCURB	0.3756 *** (5.5254)	0.443220 *** (5.8353)
LnLURB	0.0038 (0.1505)	0.009954 (0.3361)
W × lnPURB	−0.0836 (−0.4718)	0.302175 (1.6176)
W × lnEURB	−0.2815 *** (−3.2823)	−0.176597 * (−1.7838)
W × lnCURB	−0.3425 *** (−2.9246)	−0.377317 *** (−2.9382)
W × lnLURB	0.1134 ** (2.1029)	0.156938 *** (2.5811)
W × dep.var.	0.4060 *** (8.3842)	−0.236068 *** (−2.8781)
teta	NA	0.069263 *** (5.488873)
R-squared	0.9740	0.9541
corr-squared	0.1451	0.6634
Wald-spatial-lag test	35.6650 ***	35.8974 ***
LR-spatial-lag test	33.7434 ***	NA
Wald-spatial-error test	25.1706 ***	33.2562 ***
LR-spatial-error test	23.5593 ***	NA
Hausman test	Statistics	df
	130.4353 ***	9

Note: ***, **, and * denote statistical significance at 1%, 5%, and 10%, respectively. The parameters in parentheses are the t statistics, NA means no relevant data, and the intercept terms of all models are not shown in the table.

**Table 18:** Direct Effects, Indirect Effects and Total Effects.

	Direct Effects	Indirect Effects	Total Effects
LnPURB	0.4223 ***	0.1274	0.5497 **
LnEURB	0.2017 ***	−0.2973 **	−0.0956
LnCURB	0.3526 ***	−0.2970 *	0.0556
LnLURB	0.0178	0.1785 **	0.1964 **

Note: ***, **, and * denote statistical significance at 1%, 5%, and 10%, respectively.

## Data Availability

Not applicable.
